# Preclinical Testing of Living Tissue-Engineered Heart Valves for Pediatric Patients, Challenges and Opportunities

**DOI:** 10.3389/fcvm.2021.707892

**Published:** 2021-08-19

**Authors:** Ionela Movileanu, Marius Harpa, Hussam Al Hussein, Lucian Harceaga, Alexandru Chertes, Hamida Al Hussein, Georg Lutter, Thomas Puehler, Terezia Preda, Carmen Sircuta, Ovidiu Cotoi, Dan Nistor, Adrian Man, Bogdan Cordos, Radu Deac, Horatiu Suciu, Klara Brinzaniuc, Megan Casco, Leslie Sierad, Margarita Bruce, Dan Simionescu, Agneta Simionescu

**Affiliations:** ^1^Regenerative Medicine Laboratory, University of Medicine, Pharmacy, Science and Technology “George Emil Palade”, Târgu Mureş, Romania; ^2^Institute of Cardiovascular Diseases and Transplant, Târgu Mureş, Romania; ^3^Department for Experimental Cardiac Surgery and Heart Valve Replacement, School of Medicine, University of Kiel, Kiel, Germany; ^4^Biocompatibility and Tissue Regeneration Laboratory, Department of Bioengineering, Clemson University, Clemson, SC, United States; ^5^Aptus Bioreactors, LLC, Clemson, SC, United States; ^6^Tissue Engineering Laboratory, Department of Bioengineering, Clemson University, Clemson, SC, United States

**Keywords:** acellular scaffolds, autologous cells, bioreactor conditioning, orthotopic implantation, cell seeding

## Abstract

**Introduction:** Pediatric patients with cardiac congenital diseases require heart valve implants that can grow with their natural somatic increase in size. Current artificial valves perform poorly in children and cannot grow; thus, living-tissue-engineered valves capable of sustaining matrix homeostasis could overcome the current drawbacks of artificial prostheses and minimize the need for repeat surgeries.

**Materials and Methods:** To prepare living-tissue-engineered valves, we produced completely acellular ovine pulmonary valves by perfusion. We then collected autologous adipose tissue, isolated stem cells, and differentiated them into fibroblasts and separately into endothelial cells. We seeded the fibroblasts in the cusp interstitium and onto the root adventitia and the endothelial cells inside the lumen, conditioned the living valves in dedicated pulmonary heart valve bioreactors, and pursued orthotopic implantation of autologous cell-seeded valves with 6 months follow-up. Unseeded valves served as controls.

**Results:** Perfusion decellularization yielded acellular pulmonary valves that were stable, no degradable *in vivo*, cell friendly and biocompatible, had excellent hemodynamics, were not immunogenic or inflammatory, non thrombogenic, did not calcify in juvenile sheep, and served as substrates for cell repopulation. Autologous adipose-derived stem cells were easy to isolate and differentiate into fibroblasts and endothelial-like cells. Cell-seeded valves exhibited preserved viability after progressive bioreactor conditioning and functioned well *in vivo* for 6 months. At explantation, the implants and anastomoses were intact, and the valve root was well integrated into host tissues; valve leaflets were unchanged in size, non fibrotic, supple, and functional. Numerous cells positive for a-smooth muscle cell actin were found mostly in the sinus, base, and the fibrosa of the leaflets, and most surfaces were covered by endothelial cells, indicating a strong potential for repopulation of the scaffold.

**Conclusions:** Tissue-engineered living valves can be generated *in vitro* using the approach described here. The technology is not trivial and can provide numerous challenges and opportunities, which are discussed in detail in this paper. Overall, we concluded that cell seeding did not negatively affect tissue-engineered heart valve (TEHV) performance as they exhibited as good hemodynamic performance as acellular valves in this model. Further understanding of cell fate after implantation and the timeline of repopulation of acellular scaffolds will help us evaluate the translational potential of this technology.

## Introduction

The most frequent pathology in the pediatric population is represented by congenital involvement of the right ventricular outflow tract, accounting for 20% of the total number of cardiac congenital malformations (1). Present alone or associated with other tissues, pulmonary valve stenosis is ranked third in the list of most frequent congenital cardiac conditions (2). For pulmonary valve replacement in this age category group, biological prostheses are preferred, those including the classical glutaraldehyde fixed, cryopreserved homograft bioprostheses, decellularized homografts, or mechanical valves (3). Artificial mechanical valves have the downside of requiring lifelong anticoagulation, whereas the biological valves are characterized by a limited lifespan and functionality, especially in children (4). Heart valve replacement in pediatric patients is a challenging endeavor; in addition to the technical surgical complexity, current valve prosthesis models cannot grow with the somatic growth of the patient and thus require multiple open-heart reinterventions until adulthood (5).

The field of regenerative medicine and tissue engineering strives to develop the next generation of heart valve substitutes, i.e., living-tissue-engineered heart valves (living TEHVs), which could overcome the current available drawbacks of artificial prostheses. Having their own metabolism, TEHVs are expected to have the capacity to grow and develop simultaneously with the body, with no need for anticoagulation and minimal long-term degeneration (6). Essentially composed of scaffolds and cells (7), manufacturing this new type of heart valve substitute represents a research task involving multidisciplinary teams and several challenges. Studies of synthetic (polymeric) scaffolds seeded with endothelial cells and myofibroblasts (8) showed that the presence of repopulating cells was associated with valvular insufficiency secondary to cusps remodeling and retraction. Scaffolds seeded with autologous stem cells (9), or just endothelial cells (10), also revealed multiple drawbacks, a major one being the fibrosis and contraction of the cusps, induced by the seeded cells (9, 11).

Recently, a switch in the traditional paradigm has occurred, where scientists implanted degradable or no degradable scaffolds and used the host body as a living bioreactor, repopulating the scaffold with autologous cells after implantation. In animal models, these scaffolds performed well for 4–6 months, fulfilling their mechanical function. Currently, ongoing clinical trials investigating the behavior of decellularized pulmonary valves implanted in humans are ongoing (12), as well as heart valve replacements with other acellular scaffolds (13). These scaffolds are developed by removal of all antigenic cells, using detergents. They are based on an extracellular matrix composed of collagen and elastin fibers, which are not entirely intact (collagen peptides are released); therefore, after implantation, they could be a target for proteases from infiltrating cells, especially under adverse mechanical and biochemical conditions. In order to create a living heart valve, able to not only accomplish the critical mechanical function but also maintain the matrix homeostasis and respond to stimuli in physiological and/or pathological environments, we developed a pulmonary valve based on a decellularized scaffold seeded with fibroblasts and endothelial cells (ECs) derived from autologous adipose tissue-derived stem cells. Our hypothesis is that a living-TEHV with a controlled composition at cellular and extracellular level will accomplish its mechanical function similarly to the native valve. Our TEHV technology includes three elements: (1) decellularized valve scaffolds as the most suitable biological, structural, and functional component; (2) autologous cells seeded in the appropriate tissue layers for matrix homeostasis; (3) dynamic conditioning in bioreactors before implantation for cell adaptation to physiologic stress.

**Figure 1:**
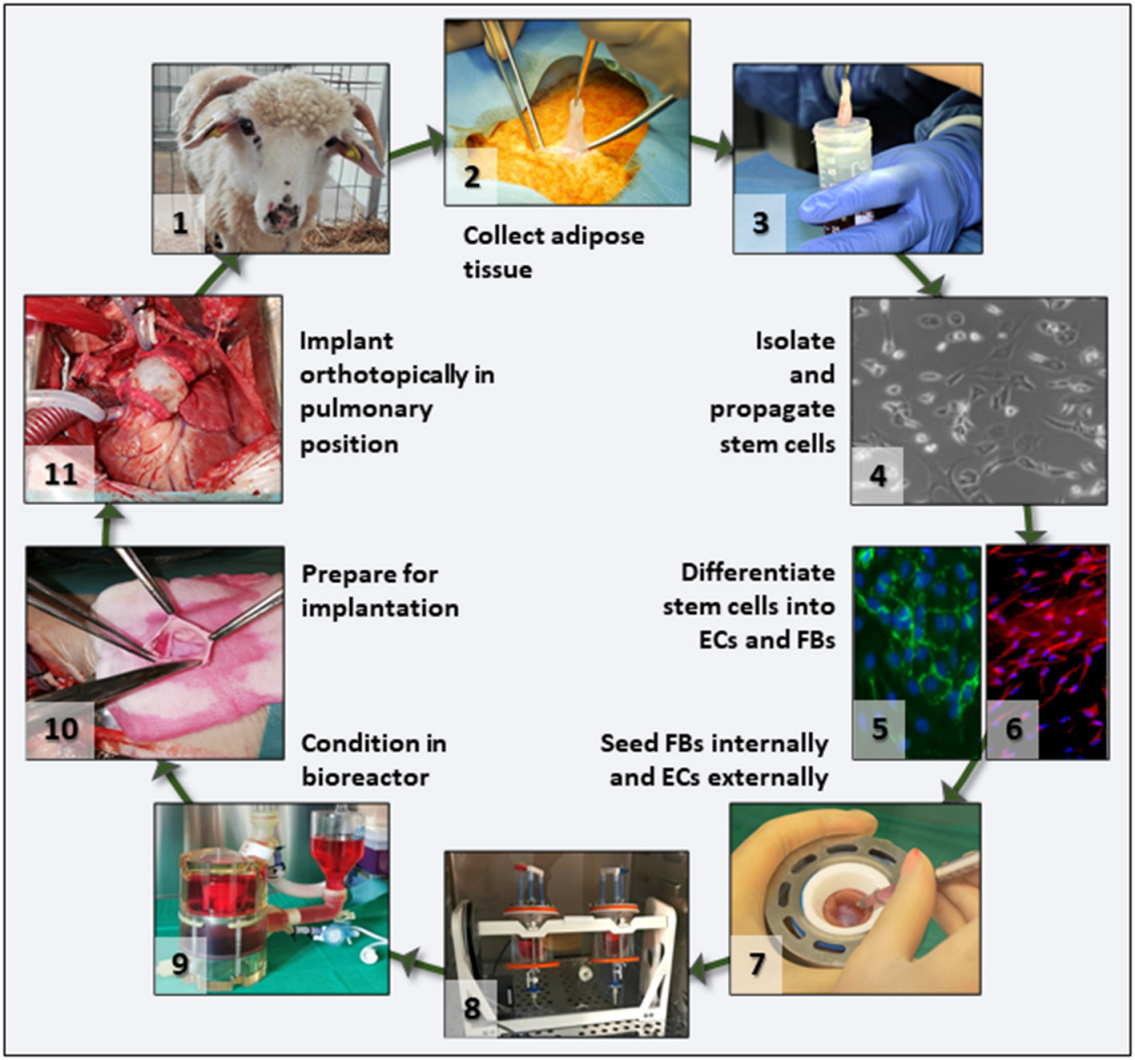
Project overview. Flowchart depicting the main translational scenario steps that were tested in this study. After animal selection **(1)**, adipose tissue was collected under aseptic conditions **(2)**, the tissue transported to the lab in culture medium **(3)**, and stem cells isolated and propagated **(4)**. For differentiation, cells were split in two batches and separately differentiated into ECs **(5)** and FBs **(6)**. Then FBs and ECs were used to seed the acellular valve scaffolds **(7)** by injection and rotation **(8)**. Seeded valves were subjected to BR conditioning in sterile media **(9)** and transported to the operating theater where they were prepared **(10)** and then implanted orthotopically as autologous implants **(11)** in the same animal from which the stem cells had been collected.

To test our hypothesis, we proposed the preclinical validation of a scenario with significant translational potential (Figure 1). Briefly, this involves *in vitro* repopulation of decellularized pulmonary valves by seeding with autologous fibroblasts (FBs) and ECs derived from adipose tissue-derived stem cells, preconditioning of the living valves in dedicated pulmonary heart valve bioreactors, and orthotopic implantation in sheep with 6 months follow-up. Each step of this scenario is described in detail, documented by results, and complemented by discussion of the challenges encountered and opportunities offered by the study.

## Materials and Methods

### Scaffolds

Ovine hearts were collected immediately after euthanasia at a local abattoir. Animals were represented by mature sheep (male and female, aged 1.5–2.0 years, weighing 50–60 kg). The harvesting technique consisted in maintaining the pericardial sack, ascending aorta, and the pulmonary trunk intact. Following harvesting, hearts were transported on ice to the laboratory for dissection.

The ovine pulmonary valves (*n* = 15) were dissected and extracted with sterile basic surgical tools, preserving 3–4 cm of the circumferentially oriented myocardium at the valve base. Distally, the arterial tissue was sectioned at the level of the pulmonary trunk bifurcation (about 5–6 cm height from the cusps commissure). After rinsing, valve roots were placed in the MechAnnulus^TM^ mounting rings by clamping the myocardium between interlocking metal rings and set in the decellularization system (PDCell^TM^ Aptus Bioreactors, LLC, Clemson, SC, USA) in batches of five roots per system. The pulmonary trunk was cannulated with Luer-barbed adaptors and connected to the perfusion system. The mounting rings are unique in the fact that they provide a no-touch mounting system for valve roots, which does not affect cusp or artery integrity. The decellularization system was perfused by a peristaltic pump with a 30-s off/3-min on cycle over 12 days under a pressure gradient of 4 mmHg (between the intraluminal and the external environment), the last step ending in sterile conditions. Studies performed previously pointed to improved cell removal from the valve roots when using perfusion methods in comparison to only immersion of the heart valves (14, 15). The decellularization procedure started with 24 h of 0.02% NaN_3_ in H_2_O (Sigma-Aldrich Chemistry, St. Louis, MO, USA) followed by 1 h perfusion of 0.05M NaOH (Lach-Ner, Neratovice, Czech Republic) and rising with H_2_O. Three batches of freshly prepared decellularization solution containing 0.05% sodium dodecyl sulfate (Sigma-Aldrich Chemistry, St. Louis, MO, USA), 0.5% TRITON X-100 (PanReac AppliChem, Castellar del Vallès, Spain), 0.5% sodium deoxycholate (Sigma-Aldrich Chemistry, St. Louis, MO, USA), 0.2% ethylenediaminetetraacetic acid (PanReac AppliChem, Spain) in 10 mM hydroxymethyl aminomethane TRIS (Sigma-Aldrich Chemistry, St. Louis, MO, USA) were circulated for 48 h each, followed by enzymatic treatments with 720 mU/ml deoxyribonuclease (PanReac AppliChem, Spain) and 720 mU/ml ribonuclease (PanReac AppliChem, Spain) for 48 h, at 37°C, applied twice. In the last day of the protocol, valves were treated with 70% ethanol and then 2 h with 0.2% peracetic acid for sterilization (Merck KgaA, Darmstadt, Germany) and storage in sterile phosphate-buffered saline (PBS) at 4°C. Further details can be found in our previous publication (14, 15).

To validate the extent of cell removal, the scaffolds underwent quality tests as follows. Histology with 4′,6-diamidino-2-fenilindol (DAPI) nuclear staining on cryosections (Thermo Fisher, Waltham, MA, USA) and hematoxylin/eosin assessed the extracellular matrix architecture and integrity and the absence of cells nuclei. DNA was also extracted from *n* = 6 tissue samples from each decellularization batch, quantified by NanoDrop spectrophotometry and analyzed by EthBr agarose gel electrophoresis, as described before (15). All valves underwent sterility testing as follows: decellularized valve fragments were collected in aseptic conditions, immersed in approximately 3 ml sterile saline, in sterile 15-ml tubes, and transported to the Microbiology Department of UMFST for further processing. Culture-based methods were used for testing the sterility of the valve fragments. For this, each sample was thoroughly vortexed in 0.5 ml sterile saline, and 10 μl was dispersed on blood agar plates (Oxoid, Hampshire, UK) to isolate the potential colonizers. Afterwards, each fragment was immersed in 4 ml Muller–Hinton broth (Oxoid, Hampshire, UK) for enrichment of potentially viable bacteria. The inoculated culture media were incubated at 37°C for 18–24 h in normal atmosphere. The development of colonies and cloudy appearance of the broth followed, respectively, which indicated bacterial colonization. For each batch of valve fragments, the saline solution used for transportation was also tested for potential bacterial contamination, after mixing in 1:1 proportion with 2 × Muller–Hinton broth and incubation at 37°C. A negative control consisting of Muller–Hinton broth only was also used. The eventual colonies that grew on blood agar were isolated. If the broth presented cloudiness, 1 μl was dispersed on blood agar, to isolate the enriched bacteria. The genus or species of the isolated bacteria was identified using routine bacteriological methods (microscopy and biochemical tests). Antibiograms were performed according to EUCAST standards, for epidemiological and infection control purposes.

### Cells

#### Adipose-Derived Stem Cells

Subdermal ovine adipose tissue of about 2 cm^3^ was harvested from each sheep from the paravertebral region in an aseptic environment and sterile conditions, in the experimental station of the university. The tissue was quickly immersed in sterile cell culture media composed of Dulbecco's modified Eagle's medium (DMEM) (Sigma-Aldrich Chemistry, St. Louis, MO, USA), 10% fetal bovine serum (Biowest, Nuaille, France), and 1% antibiotic and antimycotic (Sigma-Aldrich Chemistry, St. Louis, USA) at 37°C. Using chemical, enzymatic, and mechanical agents, stem cells were isolated using a method previously described (16). Adipose-derived stem cells (ADSCs) were cultivated in flasks with culture media, in an incubator (37°C, 5% CO_2_), and propagated up to passage 3. To extend their usage and availability, the cells underwent cryopreservation in 10% dimethyl sulfoxide in 70% DMEM/20% FBS media cryotubes, at a ratio of 2 × 10^6^ cells/vial, first frozen at 2–3°C/h down to −80°C in a Corning CoolCell^TM^ device, followed by storage at −140°C in a dedicated freezer. Thawing tests followed by cell culture revealed over 85% viability of ADSCs after cryopreservation (17). After thawing, the stem cells were divided into two equal batches and differentiated toward ECs and separately toward FBs before seeding.

#### Differentiation Toward Endothelial Cell Lineage

To recreate the endothelium, the ADSCs obtained from each animal underwent a chemical and mechanical protocol with minor modifications (18). The ADSCs were cultured on gelatin-coated culture flasks and fed with differentiation media composed of Media 199 (Sigma-Aldrich, St. Louis, MO, USA), 13% fetal bovine serum, 12 ml/L antibiotics, 0.1 g/L L-glutamine (Fisher Scientific, Waltham, MA, USA), and 7.5 U/ml heparin (Sigma-Aldrich, St. Louis, MO, USA) for 1 week at 37°C, 5% CO_2_. At the beginning of the second week, the media were supplemented with Endothelial Cell Growth Supplement (ECGS, from EMD Millipore, Burlington, NA, USA), at a concentration of 50 μg/ml and cells fed for an additional 3 weeks, with passaging of the cells at a ratio of 1:3, when 80% confluence was reached. The last week of the differentiation protocol consisted in applying shear forces to the cells by positioning the plates on an orbital shaker at 200 rpm (approximately 12 dynes) in a 37°C, 5% CO_2_ incubator (19). Cell culture media were replaced every 3–4 days throughout the experiment. The evaluation of differentiation was assessed by immunofluorescent microscopy for specific EC markers: CD 31 (rabbit polyclonal, Abcam, Cambridge, UK), eNOS (rat polyclonal, Abcam, Cambridge, UK), and von Willebrand factor (rabbit polyclonal, Abcam, Cambridge, UK) at dilutions recommend by the antibody supplier. After differentiation, ECs were frozen in dimethyl sulfoxide (DMSO) and stored at −140°C until ready to seed.

#### Differentiation Toward Fibroblast Cell Lineage

ADSCs differentiation toward FBs was achieved by supplementing the culture media with 2 ng/ml transforming growth factor-beta1 (Sigma-Aldrich, St. Louis, MO, USA) (20). The protocol took place for 3 weeks with media exchange every 3–4 days and passaging of cells at a confluence of 80%. The assessment of differentiation was performed by immunofluorescent microscopy and Western blotting for specific fibroblast cell epitopes: vimentin, Pro-4-hydroxylase, and collagen type I at dilutions recommend by the antibody supplier. After differentiation, FBs were frozen in DMSO in medium and stored at −140°C until ready to seed.

### Seeding

#### FBs Seeding

To reconstitute the main layers of the valve root, FBs were seeded internally at the cusps base and externally in the adventitia layer (**Figure 4**). First, using a 1-ml syringe, 4 × 10^6^ cells were suspended in 1 ml of culture media, and by injection, the quantity was equally distributed and introduced at the bases of the three cusps. After a period of static immersion in culture media for 4 h, the intraluminal content was separated from the external volume by placing a plastic plug at the distal arterial end of the valve (**Figure 4**). Then, the valve root, while still in the MechAnnulus^TM^ mounting rings, was placed into an acrylic jar, and 16 × 10^6^ cells were suspended in the culture media surrounding the adventitia; the jar containing the TEHV was rotated for 48 h with one rotation per minute (RPM) in a cell culture incubator to facilitate FB adherence to the adventitia.

#### ECs Seeding

ECs seeding consisted likewise of a static and a dynamic phase. First, 4 × 10^6^ cells suspended in 0.9 ml of culture media were placed in the valve cusps “pockets,” distributed equally in 0.3 ml for each cusp, followed by 4 h of static incubation. After plugging the lumen with a sterile plug, 16 × 10^6^ cells were suspended intraluminally in the valve root, and the valves were rotated at 1 rpm for 48 h in a cell culture incubator. For both steps of dynamic seeding, an air pump was attached to the rotator jars *via* a sterile inline filter, continuously enriching the culture media with air from the CO_2_ incubator.

### Bioreactor Conditioning

The *in vivo* hemodynamic conditions were replicated in the laboratory using a dedicated heart valve bioreactor (Aptus Bioreactors, Clemson, SC, USA). The TEHVs were exposed cyclically to increasing systolic and diastolic pressures and frequency over a period of 5 days, until reaching the physiological pulmonary hemodynamic regimen, as follows. After EC seeding, the plug was removed, and the distal arterial segment was sutured to the cylindrical support, stabilizing the pulmonary artery component of the valve and maintaining it in the anatomical position. The sterile bioreactor components were assembled in the sterile hood, containing the seeded valve. After introducing the culture medium, the bioreactor was placed in the cell culture incubator (37°C, 5% CO_2_), attaching the air pump to the system as described above with the rotator. The Aptus Physio^TM^ software was initiated, and the starting systolic pressure was set to 10 mmHg at 17 beats per minute (BPM). On the second day, the pressure was elevated to 15 mmHg at 30 BPM followed by a pressure of 19 mmHg with 45 BPM on the third day. On the last day of conditioning, the pressure was 24 mmHg at a rate of 60 BPM and maintained overnight until surgical implantation. Two additional TEHV were prepared, following identical decellularization, seeding, and preconditioning protocols. These served as cell-seeded, non-implanted controls.

### Implantation

The animal study had obtained the approval of the University Ethics Committee of UMFST “George Emil Palade” under number 131/21.10.2016. Details of the pre- and postoperative care of the sheep implanted with TEHV have been described in detail in a recent publication (21).

Experimental animals for this study were sheep, from a local breed “Tsigai metis” produced at the Reghin Research and Development Station for Sheep and Goats, Romania.

The female sheep aged between 14 and 18 months were brought to the Experimental Station of the UMFST, and after 3 weeks of quarantine, while they were examined clinically, they were treated with ivermectin and randomly divided into two groups: Group 1, implanted with unseeded, cell-free acellular pulmonary valve scaffolds (*n* = 6), and Group 2, implanted with autologous cells-seeded pulmonary valve scaffolds (*n* = 6). The sheep in Group 2 were first used to obtain adipose tissue, as described above, and while the cells were isolated, differentiated, and seeded onto scaffolds, the animals were allowed to recover for about 3 weeks. Just before surgery, the sheep were approximately 17–19 months old and weighed around 53 ± 11 kg. Under general, inhalation anesthesia, in extracorporeal circulation, the right ventricle ejection tract was exposed through a lateral thoracotomy in the left third intercostal space. The TEHV was removed from its holder, trimmed to size, and prepared for implantation. After removal of the native pulmonary valve, the TEHV was implanted in an orthotopic pulmonary position. Its functionality was evaluated with transesophageal and epicardial ultrasound at the time of implantation and at explantation using a Mindray DC-N6 echocardiograph (Mindray, Shenzhen, China). For the transthoracic approach, the P7-3 transducer was utilized, respectively, P7-3T, for the transesophageal ultrasound examination. By two-dimensional examination, the morphology and movement of the valve components were examined, in addition to the opening and closing mechanisms. By Doppler, the competence was evaluated along with the presence of stenosis. After surgical implantation, the animals were followed up over a period of 6 months. Clinical signs were observed and recorded along with their somatic development. Periodic transthoracic ultrasound examinations were performed under mild animal intravenous sedation. At the end of the follow-up period, the sheep weighing about 70 ± 9 kg underwent transesophageal and epicardial ultrasound under general anesthesia with orotracheal intubation followed by explantation of the valves. Explants were macroscopically examined and immersed in 10% neutral buffered formaldehyde (Sigma-Aldrich, St. Louis, MO, USA) followed by embedding in paraffin. Standard histological sections were performed, and subsequently, mounting on glass slides and hematoxylin/eosin staining were realized. For immunohistochemistry, we utilized a Dako mouse antismooth muscle actin antibody clone 1A4 (M0851) at 1:100 dilution and a horseradish peroxidase (HRP) detection kit as recommended by the manufacturer (Dako, Glostrup, Hovedstaden, Denmark). For quantitative analysis, nine random images were taken at 40 × magnification from a-SMA IHC-stained slides from each group (three from the valve base, three from the mid leaflet area, and three from the tip), and the number of positive (brown) cells per high power field (HPF) was calculated and compared between groups.

### Statistics

Single-factor analysis of variance (ANOVA) was used to compare infiltrating cell numbers and echocardiographic measurements between the two TEHV groups.

## Results

### Scaffold Decellularization and Sterilization

**Figure 2:**
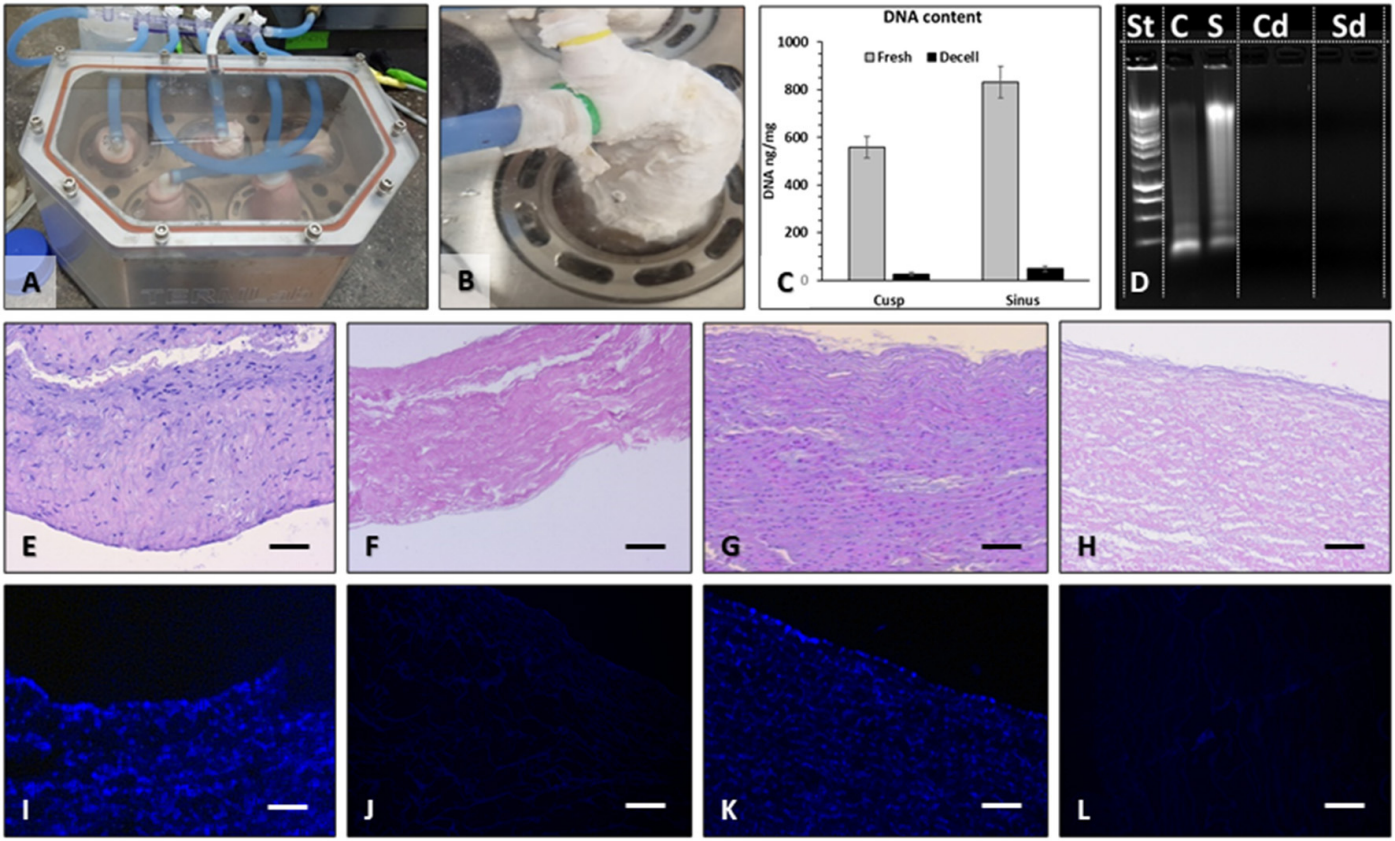
Pulmonary valve decellularization. Valves were mounted onto purpose-designed external supports and inserted into the pressurized system **(A)**, which perfused each valve individually **(B)** with detergents and enzymes. For decellularization validation, DNA was extracted from the sinus and cusp tissues and quantified by NanoDrop **(C)**, using fresh tissues as controls. Extracted DNA was also run on ethidium bromide agarose gels by electrophoresis and imaged in UV light **(D)**. St, molecular weight DNA standard ladder; C, fresh cusp; S, fresh sinus; Cd, decellularized cusp, two lanes; Sd, decellularized sinus, two lanes. Histology using H&E stain of fresh cusp **(E)**, decellularized cusp **(F)**, fresh sinus **(G)**, and decellularized sinus **(H)** and using DAPI stain for nuclei (blue) in fresh cusp **(I)**, decellularized cusp **(J)**, fresh sinus **(K)**, and decellularized sinus **(L)**. Bars in all histology images are 200 μm.

For this study, we performed nine perfusion decellularization procedures, yielding a total of 45 decellularized valves. One valve from each batch of five was randomly selected for quality control. Macroscopic evaluation of each valve at the end of the decellularization protocol did not reveal any signs of mechanical tears in any of the valve components. Decellularized valves acquired the “snow-white” color typical of acellular tissues. The sterility tests performed at the protocol finale revealed the absence of microbial contamination of all valves and of the storage media, except for four valves, which were removed from the study. Histological assessment by fluorescent staining for nuclei (DAPI) and classic optical microscopy (H&E stain) revealed preserved tissue matrix architecture and the three layers in the valve cusps, sinuses, and arterial walls along with complete absence of cell nuclei (Figure 2). These histological findings were confirmed by DNA extraction and quantitation, showing >98% reduction of DNA content and total absence of nucleic material detected by ethidium bromide agarose electrophoresis (Figure 2).

### Scaffold Revitalization With Cells

Fourteen ADSCs cultures were obtained from *n* = 14 sheep, generating a total of over 100 × 10^6^ cells. Each batch of cells was carefully labeled with the sheep number before freezing, to allow for autologous use later. To extend their availability and usability, all cells underwent cryopreservation in DMSO followed by thawing when needed for differentiation. ADSCs multipotency was confirmed earlier using specific histological stains after differentiating them toward osteogenic, adipogenic, and chondrogenic lineages using special kits (17).

#### ECs Differentiation

**Figure 3:**
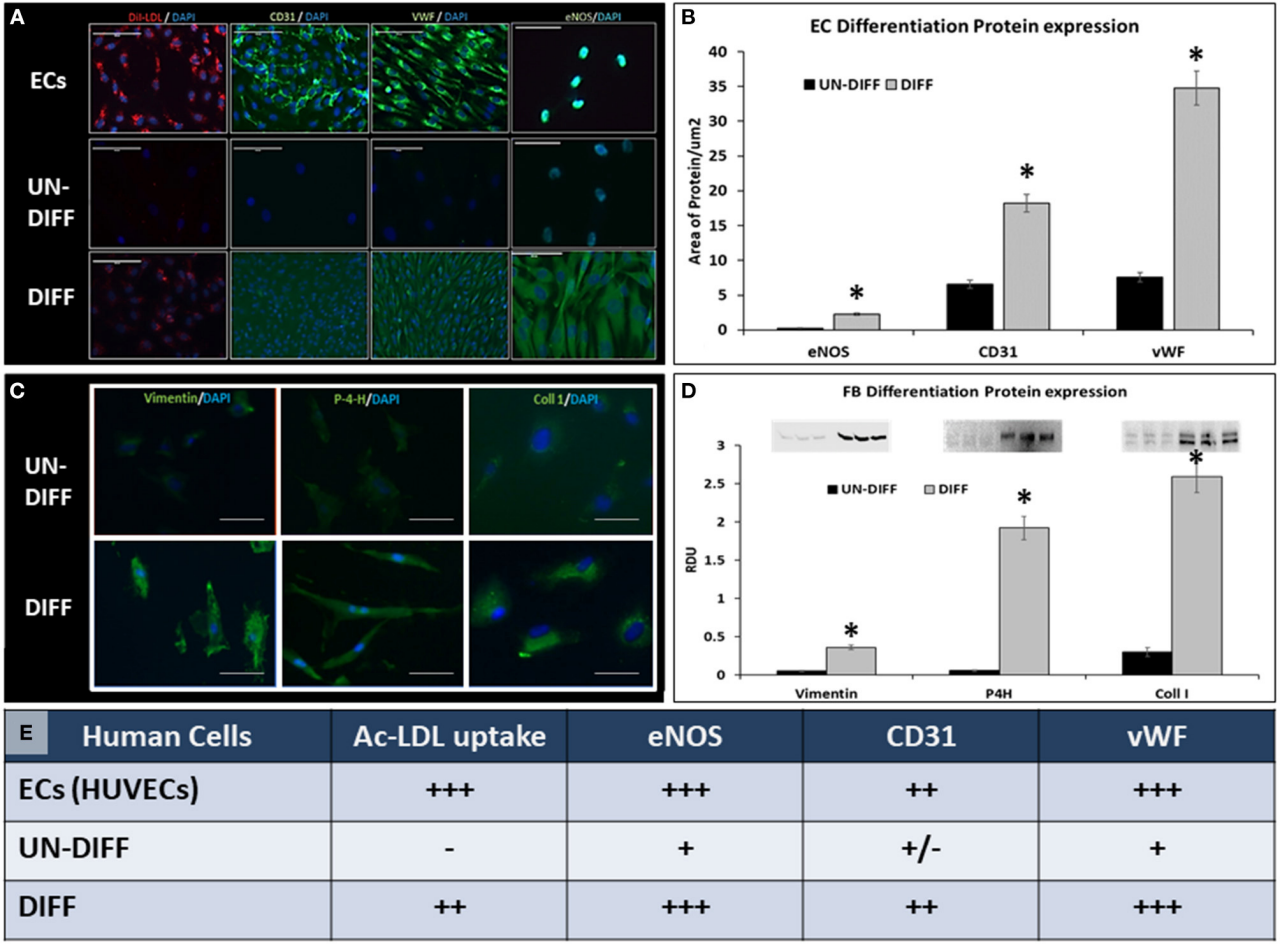
Differentiation of adipose-derived stem cells. **(A)** For differentiation into ECs, ADSCs were subjected to ECGS media followed by dynamic shear. HUVECs (ECs) served as controls. For validation, cells were incubated with DiI-labeled LDL (DiI-LDL) and imaged by fluorescence microscopy (intracellular red particles). Cells were also immunostained for CD31, VWF, and eNOS (green, positive). Nuclei were stained with DAPI. Bars are 100 μm. **(B)** amounts of positive eNOS, CD31, and VWF proteins in immunofluorescent images were assessed by semiquantitative image analysis normalized to area. For differentiation into FBs, ADSCs were exposed to low doses of TGF and stained for **(C)** vimentin, prolyl-4-hydroxylase (P-4-H), and collagen type I (Coll 1). Cells were also analyzed by Western blotting for same antigens and band intensity measured in the BioRad imager **(D)**. A semiquantitative analysis of cell differentiation is shown in **(E)**. Asterisk in Panels **(B,D)** designate statistically significant differences when compared to UN-DIFF. In all panels, UN-DIFF, undifferentiated stem cells; DIFF, stem cells that have been exposed to EC or FB differentiation conditions.

Twelve batches of ADSCs were differentiated into ECs in this study, using cell culture on gelatin-coated flasks in the presence of ECGS and shear. ADSCs exposed to these conditions displayed the ability to uptake DiI-AcLDL and were positive for CD31, vWF, and eNOS markers (Figure 3), while undifferentiated ADSCs exhibited very little reactivity for these markers and essentially did not uptake any DiI-LDL. Overall, about 68% of human ADSCs (hADSCs) stained positive for EC markers after differentiation.

#### FBs Differentiation

Twelve batches of FBs were prepared during this study, generating over 300 × 10^6^ cells. To evaluate the effectiveness of differentiation, we stained the cells for vimentin, prolyl-4-hydroxylase (P4H), and collagen type I and analyzed them by immunofluorescence (Figure 3). In addition, cells were extracted for protein and analyzed for the presence of the same markers by Western blotting. ADSCs differentiated into FBs expressed slightly upregulated levels of vimentin and significantly upregulated expression of P4H and type I collagen, while undifferentiated ADSCs expressed very low or undetectable levels of these FBs markers. Overall, about 75% of hADSCs stained positive for HSP47, P4H, and COL I.

#### Cell Seeding and Bioreactor Conditioning

**Figure 4:**
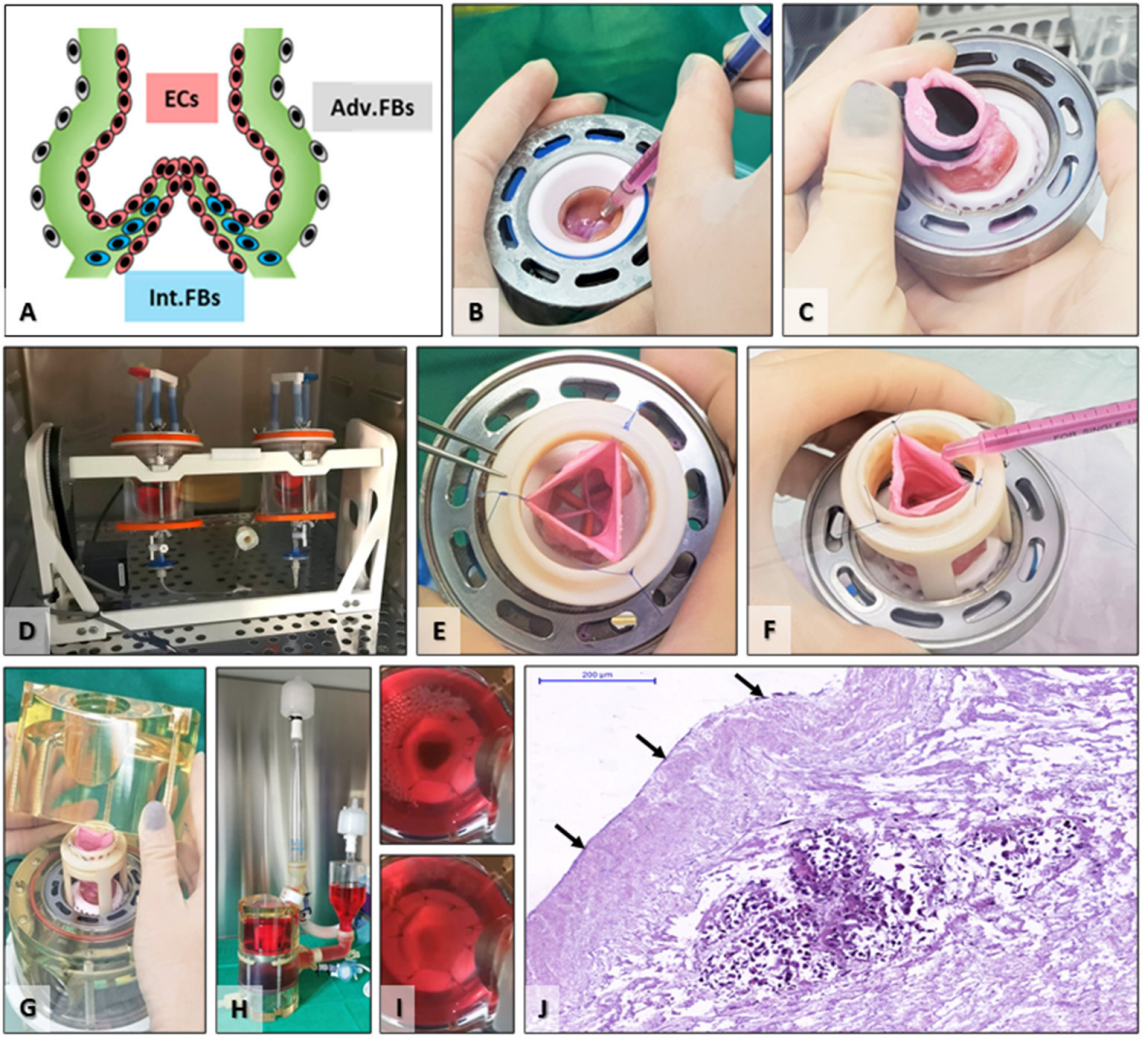
Scaffold seeding and bioreactor preconditioning. **(A)** Schematic depicting the targeted cell distribution by seeding FBs internally (Int.FBs, blue) as well as adventitially (Adv.FBs, gray) and ECs in the root lumen, on cusps, sinus, and artery surfaces (ECs, red). **(B)** FBs seeding by injection in the base of each cusp. **(C)** Placing of an aortic plug to separate the lumen from the exterior environment. **(D)** TEHVs with aortic plugs placed in FBs suspension in the acrylic seeding cups on the rotator for adventitial seeding. **(E)** After removal of the plug, the pulmonary artery was temporarily maintained open using three or more single sutures, **(E)** for intraluminal seeding of ECs **(F)**. **(G)** Positioning the seeded valve in the bioreactor, **(H)** fully assembled heart valve bioreactor just before insertion in the incubator. **(I)** still shots saved from video recordings taken during valve conditioning in the bioreactor showing open (top) and closed (bottom) positions. **(J)** H&E staining of control TEHV (seeded and preconditioned, not implanted); arrows point to ECs surface coverage, while the cell-rich island in the middle is representative of interstitially injected FBs.

The goal of seeding was to place cells in the appropriate anatomical areas and revitalize the scaffold before implantation (Figure 4). To achieve this goal, we first injected FBs in the valve cusp interstitium, then used solid plugs to separate the lumen from the adventitia, and rotated the valve in an FBs suspension for adventitial seeding. Finally, we seeded the valve lumen with a suspension of ECs on a rotator. The valves functioned well in the bioreactor, with wide opening (mean geometrical orifice area of ~1.4 cm^2^ for valves of mean external diameters of approximately 16 mm) and perfect closure (Figure 4). The control TEHVs, i.e., the seeded, conditioned but not implanted valves, were assessed by histology, which revealed the presence of FBs at the cusps base and the adventitia and ECs on the luminal surface of the valve cusps (Figure 4). Finally, seeded valves were exposed to a gradual increase in the frequency and pressure until reaching the hemodynamic regimes of pulmonary circulation. Central closure with complete opening of the three cusps was observed along with fluid movement of the sinuses and arterial walls of the valve, synchronically with the cardiac cycle's phases—systole and diastole. No signs of tears or laceration of tissue were documented during the conditioning protocol.

### Surgical Implantation and Follow-Up of TEHV

**Figure 5:**
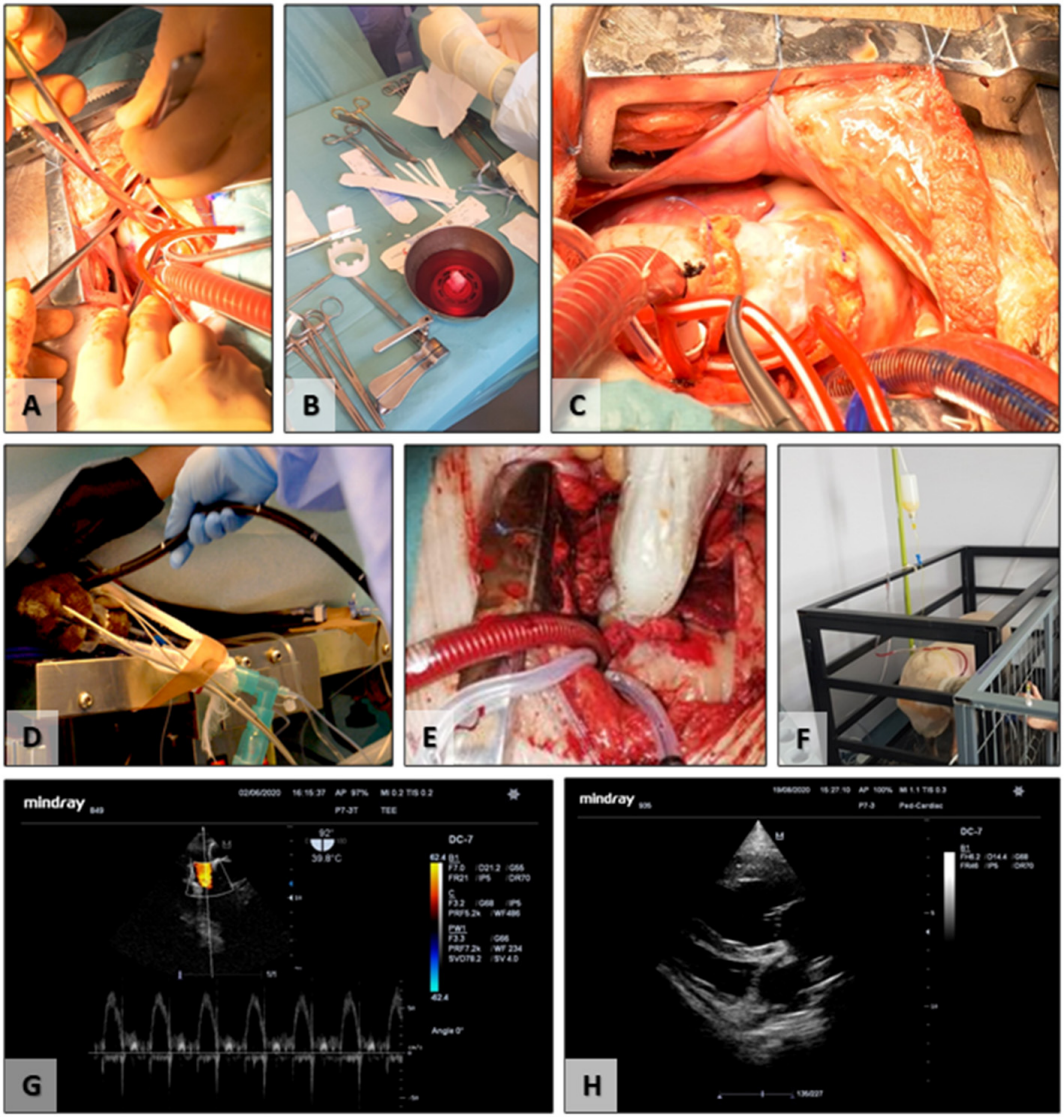
Surgical implantation and follow-up. **(A)** Removal of the native pulmonary valve; **(B)** preparation of the TEHV before implantation; **(C)** final surgical aspect, postimplantation; **(D)** insertion of the transesophageal ultrasound probe; **(E)** intraoperative epicardial ultrasound; **(F)** postoperative experimental animal transthoracic ultrasound; **(G)** transesophageal ultrasound evaluation of the TEHV; **(H)** postoperative transthoracic evaluation of the cardiac cavities and TEHV.

All animals included (*n* = 12) in the study successfully underwent the surgical implantation procedure. Animals were randomly divided into Group 1 (*n* = 6) implanted with acellular scaffolds, not seeded with cells, and Group 2 (*n* = 6) acellular scaffolds seeded with autologous FBs and ECs derived from ADSCs and conditioned in bioreactors (Figure 5). The first two valves from Group 1 were explanted early due to infective endocarditis. These were replaced by two new animals for Group 1. All surgeries were successful, with an average of 3.5 h of surgery time and with an average of 70 min of cardiopulmonary bypass, without any associated intraoperative death. In the immediate postoperative period, animals presented proper clinical status and evolution. Limited secretions were presented on the pericardial drain before removal of the tubing. Surgical incisions healed without any signs of infections or pathological accumulations. No fever or signs of acute heart failure were recorded. Restoration of vital organ function was present immediately after extubating.

Functionality evaluation of the TEHVs was periodically assessed using ultrasound for up to 6 months (Table 1). The initial evaluation was performed intraoperative using the transesophageal probe and epicardial access. The two-dimensional morphological assessment presented physiological movement of the three cusps with central cooptation and good opening.

**Table 1:** Echocardiographic evaluation of the TEHVs.

**Timeline**	**Initial evaluation—at implantation**			**End of the follow-up evaluation**		
**Animal #**	**Right and left heart morphology and function**	**TEHV morphology and function**	**Trans- TEHV maximum velocity (m/s)**	**Right and left heart morphology and function**	**TEHV morphology and function**	**Trans- TEHV maximum velocity (m/s)**
**Group 1 - control TEHVs**						
#1	Normal size and function	Normal function	0.5	Normal size and function	Trivial regurgitation	0.7
#2	Normal size and function	Normal function	0.8	Normal size and function	Moderate regurgitation	0.5
#3	Normal size and function	Normal function	0.7	Dilatation of right ventricle	Important regurgitation	0.7
#4	Normal size and function	Normal function	0.6	Normal size and function	Normal function	0.6
#5	Normal size and function	Normal function	0.5	Normal size and function	Normal function	0.7
#6	Normal size and function	Normal function	0.8	Normal size and function	Normal function	0.7
**Mean** **+/– SEM**			**0.65+/– 0.13**	**Mean** **+/– SEM**		**0.65+/−0.08**
**Group 2 - cell seeded TEHVs**						
#1	Normal size and function	Normal function	0.7	Dilated right ventricle	Important regurgitation	0.5
#2	Normal size and function	Normal function	0.8	Normal size and function	Normal function	0.7
#3	Normal size and function	Normal function	0.5	Normal size and function	Moderate regurgitation	0.6
#4	Normal size and function	Normal function	0.6	Normal size and function	Normal function	0.6
#5	Normal size and function	Normal function	0.7	Dilated right ventricle and pulmonary artery trunk	Hyper-echogenic aspect of the TEHV with impaired opening of the cusps	2.4
#6	Normal size and function	Mild regurgitation	0.7	Normal size and function	Mild regurgitation	0.7
**Mean** **+/– SEM**			**0.66** **+/– 0.10**	**Mean** **+/– SEM**		**0.91** **+/−0.73**

*Statistical analysis revealed no significant difference between the mean maximum trans-TEHV velocity between the two groups at all time points (p = 0.06)*.

Echocardiography in Group 1, control TEHVs, pointed to the presence of mediastinal collections in two out of the six animals and a pericardial effusion that was drained. Animals presented with preserved ventricular ejection fraction along with no changes in the cardiac cavities dimension excepting one animal with light dilatation of the right cardiac cavities resulting from an important valvular regurgitation. Moderate insufficiency was observed in one animal and trivial in another.

In Group 2 of cell-seeded TEHVs, five out of six animals developed non-hemodynamically significant circumferential pericardial collections, with no indication for surgical drainage. On consecutive exams in two animals, resorption of the liquid was noted. At repeated exams, two animals presented enlarged right cavities. One of these two animals additionally developed valve stenosis with a planimetric measured area of 1.2 cm^2^. In terms of regurgitation, one valve presented a moderate degree of insufficiency but with no impact on the right heart cavities. Due to technically surgical malposition (higher suture on the TEHV basis) of one TEHV, it presented mild central regurgitation. TEHV cusps revealed to be thin, supple, and mobile, with complete opening on echocardiography. Prior to the animal's euthanasia, TEHVs were examined by epicardial ultrasound.

### TEHV Explants and Their Analysis

**Figure 6:**
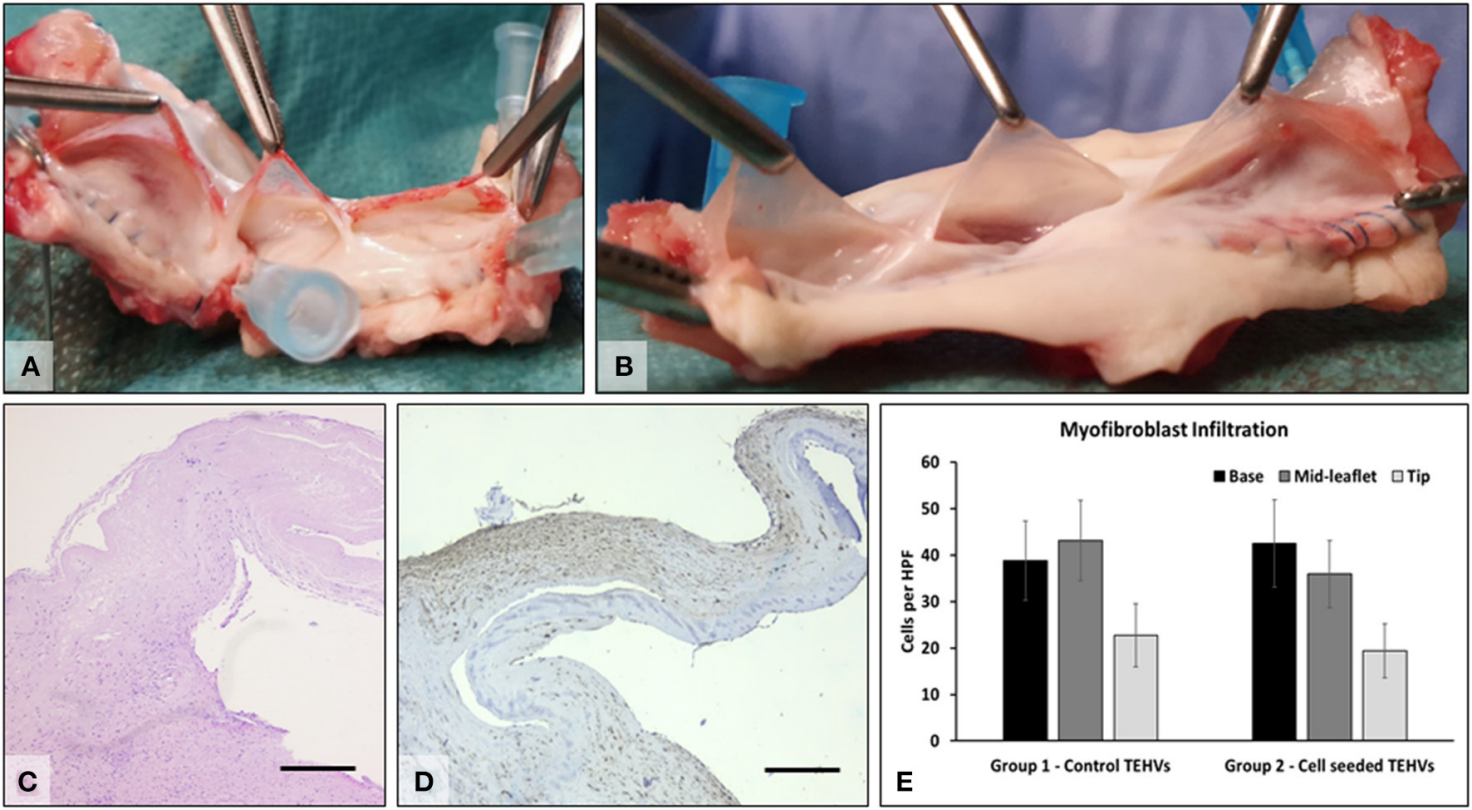
Explant analysis. **(A)** Representative macroscopic images of freshly explanted TEHV and **(B)** cell-seeded TEHV. **(C)** Representative H&E stain of cells found within the valve interstitium; **(D)** immunohistochemistry for alpha-smooth muscle actin (brown, positive). Bar is 200 μm. **(E)** Quantitative data on alpha-smooth muscle actin positive cells infiltrated in the base, middle, and tip areas of the TEHVs. Statistical analysis revealed no significant differences in the number of infiltrated cells between the two valve groups.

At 6 months after implantation, animals were prepared for euthanasia and explantation of the TEHV. At explantation, the implants and anastomoses were intact, and the valve root was well integrated into the host tissues, with a shiny endothelium covering the whole implant and the anastomoses, with no evidence of thrombi. The tubular implants looked unaltered and could hold saline if added distally, pointing to competent valves. The implants were then sectioned longitudinally, and opened roots were photographed. The valve leaflets were unchanged in size or thickness and were non-fibrotic, thin, supple, and semitransparent. Numerous cells positive for a-smooth muscle cell actin were found mostly in the sinus, base, and the fibrosa of the leaflets, indicating a strong potential for repopulation of the scaffold. Flat monolayers of endothelial cells were found on the surfaces of the valve leaflets, the sinus walls, and the pulmonary artery (Figure 6). No major differences were noted in performance or cell repopulation of cell-seeded vs. non-cell-seeded valves in the sheep study.

## Discussions

### Key Findings

In this project, we proposed a translational scenario (Figure 1) based on our previous experience with acellular valve scaffolds, which performed very well hemodynamically in the right ventricular outflow tract (RVOT) in sheep (22, 23). The decellularization protocol proved to be effective in removing all nucleic materials while preserving the integrity and architecture of the ECM, as reported earlier in pilot studies (15). Acellular valve scaffolds have several advantages: they preserve the natural 3D architecture, have excellent hemodynamics and biomechanics, are highly biocompatible, are not antigenic, maintain integrity for extended periods of time, and serve as excellent porous substrates for cell adhesion and repopulation (24, 25). Despite exposing bare collagen surfaces to flowing blood, we could not detect any thrombus structures in any of the explants, and there was no need for systemic anticoagulation. Notably, TEHVs can be considered tissue-derived bioprostheses heart valves, which, according to current clinical guidelines, require oral anticoagulation with Vitamin K antagonists for 3 months postoperation (9). In pilot studies, we explanted two acellular valves: one was explanted early (after 48 h) and a second after 7 days, both due to non-valve-related issues. As seen by histology, the valve at 48 h showed a thin layer of fibrin on the surfaces, which disappeared after 7 days (data not shown) and clearly was not present on any of the explanted valves at 6 months. This was not investigated systematically in the current study but confirms results published earlier by us and others (8, 26). Similarly to other investigators using decellularized valves (12, 27), in our hands, there was no inflammatory or immune reaction to the implant, no calcification was noted, and no pannus or tissue overgrowth was found in any of the valves analyzed in this study, indicating high biocompatibility and lack of antigenicity.

Earlier, we seeded acellular valve scaffolds with freshly isolated autologous stem cells (ADSCs) and immediately implanted them in sheep, without any stem cell differentiation or any dynamic bioreactor preconditioning; as most of the cells died within 3–4 days after implantation, we noted that stem cells were very vulnerable to dynamic functional pressures and flow (22), which was also confirmed *in vitro* using cell-seeded scaffolds tested in bioreactors (28). To overcome these issues, we hypothesized that seeding with mature cells and implementing progressive dynamic conditioning would enhance the viability of cells after implantation. Therefore, in current study, we decided to isolate the stem cells, differentiate them *in vitro* into more robust FBs and ECs, and use those cells for scaffold seeding; in addition, we subjected seeded scaffolds to progressive adaptation in heart valve bioreactors, by slowly increasing flow, frequency, and pressures so that within 5 days, the constructs reached pulmonary valve conditions. Endothelialization is a fundamental target for researchers and clinicians involved in vascular and valvular replacements (29, 30). Varied modalities to differentiate and to promote ADSCs commitment toward ECs were described, including genomic manipulation (31), immune modulation (32), induced hypoxia (33), and specific growth factors (34, 35). Comparative studies of differentiation of ADSCs toward ECs lineage revealed improved results when combinations of ECGS and shear were used (36, 37) pointing to the importance of recreating the *in vivo* conditions. Finally, in previous works, we mounted valve scaffolds in acellular pericardial tubes and implanted them as a shunt in the RVOT, which did not fully mimic the appropriate clinical application of such valves (22, 23). In the current study, we implanted the revitalized valves orthotopically using open-heart surgery under cardiopulmonary bypass (CPB), which better approximates the clinical use of such valve implants. Cell-seeded valves maintained their viability after progressive bioreactor conditioning and functioned well *in vivo* for 6 months. Cells positive for a-smooth muscle cell actin were found in the valves interstitium flat monolayers of endothelial cells were found on the surfaces indicating a strong potential for repopulation of the scaffolds.

### Conclusions

To our knowledge, this is the first study to document preclinical validation of decellularized valvular scaffolds repopulated with heart-valve-specific cells differentiated from autologous stem cells and preconditioned before implantation using a dedicated bioreactor.

Our decellularization protocol resulted in acellular and sterile heart valve scaffolds, confirmed by histological assessment, DNA quantitation, and sterility tests. Preserved valve geometry and structure were documented along with conserved matrix architecture. Scaffold functionality was further assessed by ultrasound evaluation while functioning in a bioreactor, revealing absence of valvular regurgitation (data not shown here). Ovine ADSCs proved to be a convenient source of stem cells, their cryopreservation after propagation proving to be a good option for increasing their availability. Their differentiation toward heart-valve-specific cell lineages was successful, with upregulation of major markers, albeit not reaching expressions of adult mature target cells. Seeding efficacy was not wide ranging, with areas of scaffolds remaining cell free interstitially; however, cell viability was maintained during the seeding protocol. Bioreactor preconditioning did not change the integrity of the scaffold or the seeded cells. Surgical implantation in pulmonary position was successful, with no major incidents or complications, while the animal's follow-up proved challenging due to poor transthoracic ultrasound windows and their rapid somatic growth. Notably, host cells were found populating the implanted scaffolds in large numbers, with preference for the cusp base, fibrosa, and spongiosa layers of the valve cusps. Cells were distributed homogeneously, resembling regeneration processes more that hyperplasia. Overall, we concluded that cell seeding did not negatively affect TEHV performance, as they exhibited as good hemodynamic performance as acellular valves in this model.

### Challenges and Opportunities

#### Scaffolds

Minimizing mismatch in the size of implanted valves is very important clinically (38). Initially, we worked with adult porcine valves (22), but they were too large for orthotopic implantation in the ovine pulmonary position; we tested a few valves from juvenile pigs that matched in size, but it was very difficult for us to ensure a constant supply of juvenile pigs for this study. Thus, we decided to pursue ovine tissue sources, which were a much better size match.

#### Decellularization

Removing all the cells was easily achieved using the perfusion system utilized before for aortic valve roots, with minor modifications in procedures (14), including adaptation of the mounting rings and a diminution of working pressures to less than physiological 20 mmHg. Sterility and long-term storage are a major challenge in TEHV. It is paramount that decellularization protocols efficiently remove all cells from cusps, muscle, and artery. In earlier phases of the project, we utilized peracetic acid sterilization of the valves while they were still mounted in the support rings, inside the perfusion system. We realized later that certain areas of the root tissues (clamped in the support rings) were not sterile because of lack of access to the sterilant. A few of these valves that were implanted had to be explanted early due to endocarditis. To solve this issue, we decided to take out the valves from the rings after decellularization, sterilize the entire root by immersion, and then remount them in a new set of sterile rings for further seeding and conditioning. Routine sterility testing of tissues and storage solutions is highly recommended throughout the process of manufacturing TEHV.

#### Cells

ADSCs are relatively easy to isolate and culture from subdermal adipose tissue samples. In a pilot study, we collected adipose tissue from several anatomical areas to determine the optimal source for ADSCs and found the interscapular area as most favorable (39). When needed, ADSCs can be safely cryopreserved for later use. Differentiated stem cells expressed some, but not all, specific markers for FBs or ECs; it is not clear whether this affects cell behavior after implantation. Furthermore, it is not clear if cryopreservation could be applied after ADSC differentiation into FBs and ECs. More detailed studies on ADSC-derived FBs and ECs are warranted.

#### Seeding

Manual injection in the cusp interstitium was limited to just a few sites, and it is not clear if cell migration took place later from the cell bolus. We do not know at this point if the cells found in the 6-month explants were the same cells that were seeded initially, or they were host-derived infiltrated cells. More studies with cell tracers on a discrete timeline are needed to answer this question. In addition, improved interstitial seeding methods are needed to address the need to distribute cells in specific anatomic areas more homogeneously.

#### Bioreactor

The ramping-up profile was not optimized but rather designed rationally. A time-dependent study looking at effects of different adaptation regimes on cell attachment and viability is needed. We do know that the bioreactor can maintain cell viability for up to 2 weeks (40). Maintaining valves alive in the bioreactor could be useful especially if implantation cannot occur as scheduled. Assembly of the bioreactor and mounting of the valve in sterile conditions are not trivial and require extensive preparation and attention to detail. Preassembly of the major bioreactor components (reservoirs, connecting tubes, transducers, and atrial and ventricular chambers) in the sterile hood was instrumental in reducing the time it took for rapid mounting of the living valves. In our hands, no signs of microbial contamination of the valves or the culture media were observed during bioreactor conditioning.

#### Implantation

Despite timely coordination between the tissue engineering lab and the operating room, valve preparation in the operating theater can sometimes take longer than expected, or the patient could require extra care before implantation. This raises the question of how to maintain valve viability while in the operating room, using cold storage, a temporary warm storage solution, or even a transportation bioreactor. In the current study, the lab was in the vicinity of the animal facility, which reduced transportation time to a minimum. Echocardiography in sheep was found difficult unless in the open chest, where transepicardial access allowed for excellent imaging. The heart and its structures were periodically examined through non-standard transthoracic echocardiography windows given the brisk animal's somatic development in the postoperative period and emerging distortions in the mediastinal anatomy; these aspects represented significant limitations for an adequate ultrasound evaluation. Additionally, in the immediate postoperative period, examinations were limited by postsurgical inflammation processes. Considering the above stated constraints, the transthoracic examinations were restricted to evaluation of mediastinal collections. Visualization of TEHV proceeded with morphological evaluation of valve components, interrogation with both color and pulse Doppler, and evaluation of the existence of any degree of regurgitation, stenosis, or both. Regarding the TEHVs, when they could not be visualized directly, appearance of indirect signs of valvular dysfunction such as dimension and functionality of the right and left ventricles and atria was assessed.

#### Post Explant Analysis

Histology on unimplanted decellularized tissues is sometimes tricky. The classical stains do not always bind efficiently to decellularized tissue components, and elastin autofluoresces intensely after decellularization. We found that washing of detergent-decellularized tissues with 70% ethanol before histological processing improved outcomes of histological analysis. Histology on explanted tissue, however, worked out much better, presumably because of host cells and protein infiltration. Immunohistochemistry was also found difficult at times because of paucity of antisheep antibodies and, at times, the diaminobenzidine reagent bound to collagen in decellularized tissues in the absence of any antibodies.

### Future Directions for Research

Further work needs to be performed to fine-tune the different aspects of this translational scenario. Stem cells could be obtained from other sources, including induced pluripotent stem cells (iPSCs), and these could be differentiated *in vitro* into the desired target cells. Seeding devices capable of inserting cells in the appropriate sites are currently being designed and tested in our group. Seeded cells should be labeled by a permanent tag that would be stable and transmissible to daughter cells and monitored *via* a convenient imaging technique. This type of study should be implemented together with a more detailed long-term investigation with more numerous time points, looking at the fate of TEHV implanted in juvenile sheep and how they adapt to somatic growth. This should be accompanied by more reliable imaging follow-up; investigation of blood parameters of inflammation, immune reactions, and coagulation; and precise assessment of the ability of the TEHV to grow with the somatic growth of the patient. To assess growth, markers should be placed strategically in different areas of the implant, and tissue thickness should be measured at all time points. A comparison of cell-seeded scaffolds with cell-free scaffolds will allow to draw important conclusions relative to the need for *in vitro* repopulation before implantation, currently a topic of controversy. Several groups are investigating cell-free approaches, relying on the body's ability to repopulate or regenerate scaffolds with the appropriate cells (41, 42); however, it is not clear at the moment whether this response is a true regenerative process or a fibrotic healing reaction. We also do not know if implantation of a cell-seeded TEHV would stimulate or suppress local regenerative and/or fibrotic processes. Overall, we believe that with our approach, we are getting closer to the target, and with further interrogation, successful implementation of TEHV will benefit pediatric patients and young adults.

## Data Availability Statement

The raw data supporting the conclusions of this article will be made available by the authors, without undue reservation.

## Ethics Statement

The animal study was reviewed and approved by University Ethics Committee of UMFST George Emil Palade under number 131/21.10.2016.

## Author Contributions

KB, GL, ThP, AS, and DS: concept, data analysis, and manuscript. IM, TeP, AM, MC, LS, MB, and AC: scaffold development, cell culture, and bioreactors. MH, HuA, LH, HaA, CS, DN, BC, RD, HS, and OC: animal surgery and explant analysis. All authors contributed to the article and approved the submitted version.

## Conflict of Interest

LS was employed by the company Aptus Bioreactors. The remaining authors declare that the research was conducted in the absence of any commercial or financial relationships that could be construed as a potential conflict of interest.

## Publisher's Note

All claims expressed in this article are solely those of the authors and do not necessarily represent those of their affiliated organizations, or those of the publisher, the editors and the reviewers. Any product that may be evaluated in this article, or claim that may be made by its manufacturer, is not guaranteed or endorsed by the publisher.
